# Mechanisms of Hypercapnia-Induced Endoplasmic Reticulum Dysfunction

**DOI:** 10.3389/fphys.2021.735580

**Published:** 2021-11-19

**Authors:** Vitalii Kryvenko, István Vadász

**Affiliations:** ^1^Department of Internal Medicine, Justus Liebig University Giessen, Universities of Giessen and Marburg Lung Center (UGMLC), Member of the German Center for Lung Research (DZL), Giessen, Germany; ^2^The Cardio-Pulmonary Institute (CPI), Giessen, Germany; ^3^Institute for Lung Health (ILH), Giessen, Germany

**Keywords:** hypercapnia, carbon dioxide, endoplasmic reticulum, protein folding, unfolded protein response

## Abstract

Protein transcription, translation, and folding occur continuously in every living cell and are essential for physiological functions. About one-third of all proteins of the cellular proteome interacts with the endoplasmic reticulum (ER). The ER is a large, dynamic cellular organelle that orchestrates synthesis, folding, and structural maturation of proteins, regulation of lipid metabolism and additionally functions as a calcium store. Recent evidence suggests that both acute and chronic hypercapnia (elevated levels of CO_2_) impair ER function by different mechanisms, leading to adaptive and maladaptive regulation of protein folding and maturation. In order to cope with ER stress, cells activate unfolded protein response (UPR) pathways. Initially, during the adaptive phase of ER stress, the UPR mainly functions to restore ER protein-folding homeostasis by decreasing protein synthesis and translation and by activation of ER-associated degradation (ERAD) and autophagy. However, if the initial UPR attempts for alleviating ER stress fail, a maladaptive response is triggered. In this review, we discuss the distinct mechanisms by which elevated CO_2_ levels affect these molecular pathways in the setting of acute and chronic pulmonary diseases associated with hypercapnia.

## Introduction

Carbon dioxide (CO_2_) is a metabolic product of cellular oxidative respiration, and is primarily eliminated from the blood and tissues by the lungs under physiological conditions. An elevation in CO_2_ partial pressure in arterial blood over 45 mmHg is termed hypercapnia. Increased CO_2_ levels are often observed in conditions where an impairment of the alveolar-capillary barrier function or a decline in alveolar ventilation occurs (Vadasz et al., 2012b; Herold et al., 2013). Various acute and chronic lung diseases, such as acute respiratory distress syndrome (ARDS), chronic obstructive pulmonary disease (COPD), asthma and cystic fibrosis are frequently accompanied by hypercapnia (Vadasz et al., 2012b; Radermacher et al., 2017). Furthermore, elevated CO_2_ levels and intermittent hypoxia combined with hypercapnia play a role in the pathogenesis of obstructive sleep apnea, atherosclerosis and obesity (Kikuchi et al., 2017; Imamura et al., 2019; Xue et al., 2021).

It is increasingly evident, that various non-excitable cells, such as alveolar epithelial cells, fibroblasts and immune cells are sensitive to the changes in CO_2_ concentrations independently of intra- and extracellular pH, reactive oxygen species (ROS) and involvement of the carbonic anhydrases (Putnam et al., 2004; Shigemura et al., 2017; Cummins et al., 2019). In contrast to earlier reports, which suggested that hypercapnia might be tolerated or even beneficial in the setting of critically ill patients (Fuller et al., 2017; Roberts et al., 2018); more recent studies have shown that elevated CO_2_ levels are associated with higher complication rates, increased risk of exacerbations, more severe disease states, worse outcomes and an increased risk of mortality both for acute and chronic lung diseases (Yang et al., 2015; Nin et al., 2017; Husain-Syed et al., 2020; Shigemura et al., 2020). In addition, translational studies established that high CO_2_ levels impair alveolar fluid clearance, innate immunity and cellular host defense, decrease cytokine production, downregulate phagocytosis and macrophage activity. Hypercapnia also stimulates nitric oxide (NO) production, therefore negatively impacting on pulmonary metabolism, aggravates epithelial cell repair, alters cellular lipid metabolism, decreases muscle anabolism, increases smooth muscle airway contractility and muscle catabolism, thus contributing to disease states and impaired recovery (Lang et al., 2000; Vadasz et al., 2008; Gates et al., 2013; Jaitovich et al., 2015; Kikuchi et al., 2017; Shigemura et al., 2018; Korponay et al., 2019). In addition, recent studies suggest that elevated CO_2_ levels increase mortality in animal models of acute lung injury secondary to viral and bacterial insults (Gates et al., 2013; Casalino-Matsuda et al., 2020).

Protein transcription, translation, folding, and maturation continuously occur in each living cell and are essential for normal physiological function. In the cell, approximately one-third of the proteome and most of the secretory and membrane proteins are processed through the endoplasmic reticulum (ER) (Brodsky and Skach, 2011). In addition, the ER regulates lipid metabolism and serves as an intracellular calcium store (Hetz et al., 2015; Schwarz and Blower, 2016). The ER coordinates numerous co- and post-translational protein modifications, including N-linked glycosylation, formation of disulfide-bonds, sequence cleavage, chaperone-assisted protein folding, recognition and targeting of the ER-localized proteins for degradation (Ellgaard and Helenius, 2003; Araki and Nagata, 2011; Ellgaard et al., 2018). Numerous ER-resident chaperons, such as calnexin, calreticulin and binding immunoglobulin protein (BiP) orchestrate co-translational folding/refolding of nascent proteins. In addition, these chaperons play a central role in the removal of terminally misfolded proteins *via* ER-associated degradation (ERAD) and are key players of unfolded protein response (UPR) during ER stress (Hebert and Molinari, 2007; Halperin et al., 2014). Up to date, three main UPR pathways, named by ER-localized proteins have been characterized: inositol-requiring enzyme 1 (IRE1), protein kinase RNA-activated (PKR)-like ER kinase (PERK), and activating transcription factor-6 (ATF6). An increase of misfolded/unfolded proteins in the ER leads to dissociation of BiP from ER stress sensors, autophosphorylation of the sensors and subsequent activation of UPR (Wang and Kaufman, 2016; Almanza et al., 2019).

Of note, ER stress plays a pivotal role in the pathomechanism of various respiratory diseases, including but not limited to COPD (and in particular cigarette smoke exposure), viral and bacterial pneumonia, asthma, interstitial lung diseases and cystic fibrosis (Korfei et al., 2008; Lawson et al., 2011; Kenche et al., 2013; Kim et al., 2013; van ’t Wout et al., 2015; Lee et al., 2016; Marciniak, 2017; Tang et al., 2017; Schmoldt et al., 2019), many of which are accompanied by hypercapnia (Vadasz et al., 2012b; Shigemura et al., 2020). Notably, these disease states also often lead to hypoxia. Indeed, low oxygen levels have also been shown to negatively impact ER homeostasis, thus inducing ER stress (Chipurupalli et al., 2019; Bradley et al., 2021). Although the effects of hypoxia on the ER lie beyond the scope of the current manuscript, it is increasingly evident that hypoxia negatively affects ER function in alveolar epithelial cells and macrophages in the lung. These effects involve the downregulation of metabolic processes and disruption of the ER chaperone activity, which result in activation of key elements of the UPR, such as PERK, eIF2a, and IRE1α (Burman et al., 2018; Delbrel et al., 2018, 2019; Diaz-Bulnes et al., 2019; Bradley et al., 2021). Another cellular organelle that is tightly related to the ER is the peroxisome (Dimitrov et al., 2013). Of note, recent publications suggest that hypercapnia affects peroxisome signaling by modulation of the activity and expression of peroxisome proliferator-activated receptors (Huang et al., 2016; Kikuchi et al., 2017). At the molecular level, elevated CO_2_ has been shown to activate kinases and proteins that are known to regulate ER function and/or participate in UPR, such as c-Jun N-terminal kinase (JNK), extracellular signal-regulated kinase (ERK1/2), AMP-activated protein kinase (AMPK), B-cell lymphoma 2 (Bcl-2), and caspase-7 (Vadasz et al., 2008, 2012a; Welch et al., 2010; Casalino-Matsuda et al., 2015; Dada et al., 2015; Shigemura et al., 2018). Furthermore, recent reports suggest that CO_2_ can impact post-translational protein biochemistry by carbamate formation and subsequent protein carbamylation (Meigh et al., 2013; Linthwaite et al., 2018). In this review, we will focus on the molecular mechanisms by which hypercapnia impairs protein folding in the ER. Unfolding/misfolding of proteins in the ER by elevated CO_2_ levels result in enhanced protein retention or degradation, thereby impairing subsequent protein trafficking, and thus overall cellular and tissue function.

## Hypercapnia and Endoplasmic Reticulum Homeostasis

It is well documented that protein maturation in the ER requires a specific milieu, including high Ca^2+^ levels, sufficient amounts of ATP, and an appropriate oxidizing environment (Jager et al., 2012; Almanza et al., 2019). In particular, in the past two decades, a number of studies revealed that disruption of the ER folding environment leads to accumulation of misfolded/unfolded proteins, induces ER stress and subsequent activation of the UPR (Araki and Nagata, 2011; Wang and Kaufman, 2016).

### Elevated CO_2_ Levels, Cellular ATP and Endoplasmic Reticulum Redox Balance

Protein translation and subsequent post-translational modification of ER-resident proteins are among the highest energy consuming cellular processes (Wieser and Krumschnabel, 2001). These ER processes, including folding, translocation, quality control and UPR require energy in form of ATP. The source of ATP depends on the cellular metabolic state. ATP is generated either by oxidative phosphorylation or by glycolysis (Depaoli et al., 2019). When ATP is derived from active mitochondrial respiration (oxidative phosphorylation), the ATP molecules are possibly transferred directly into the ER through mitochondria-associated ER membrane (MAM) sites (Depaoli et al., 2019; Fan and Simmen, 2019). However, when glycolysis is the major source of cellular energy, ATP enters the ER directly from the cytosol (Depaoli et al., 2019). Of note, most of the ER-folding chaperons of the HSP70 and HSP90 protein families are ATP-dependent, and thus require energy for proper function (Sala et al., 2017). A decline in the ER ATP levels has been shown to impair disulfide bond formation, negatively impacts protein glycosylation and provokes inappropriate calcium signaling (Bravo et al., 2013).

Several studies have demonstrated that hypercapnia aggravates cellular ATP production. For example, in a recent publication it was shown that epithelial and mesenchymal cells exposed to elevated CO_2_ levels exhibit mitochondrial dysfunction and decreased ATP production (Vohwinkel et al., 2011). The reduction in ATP levels is induced by CO_2_-dependent upregulation of miR-183, which in turn downregulates expression of isocitrate dehydrogenase 2 (IDH2), a key enzyme involved in the tricarboxylic acid (TCA) cycle. This inhibition of the TCA cycle impairs mitochondrial and thus metabolic function and leads to downregulation of cellular proliferation. Importantly, these deleterious effects of hypercapnia can be rescued by application of α-ketoglutaric acid (α-KG), an intermediate metabolite in the TCA cycle, or by overexpression of IDH2, further highlighting the central role of the impeded TCA cycle in the hypercapnia-induced metabolic dysfunction (Vohwinkel et al., 2011). In line with these findings, exposure of primary human airway epithelial and lung endothelial cells to hypercapnia has been shown to attenuate mitochondrial membrane potential, decrease ATP production, and induce mitochondrial dysfunction, thus decreasing reparative potential of the cell (Fergie et al., 2019).

**FIGURE 1:**
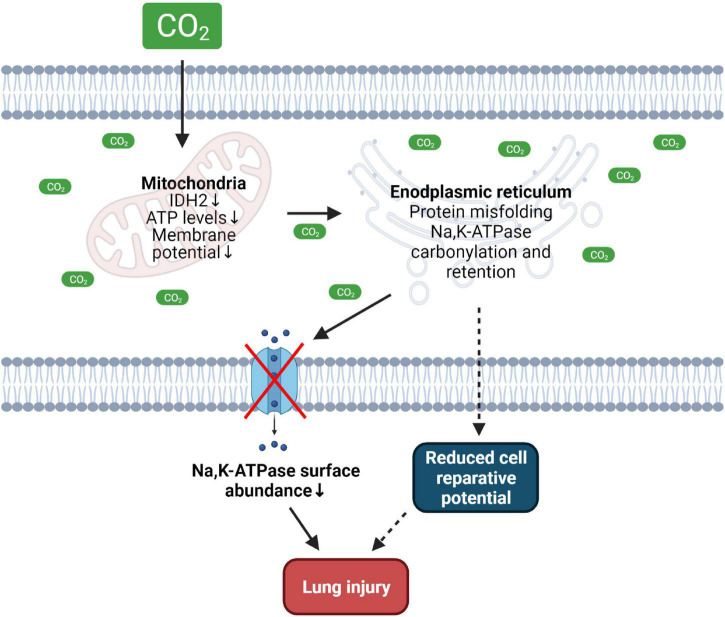
Effects of hypercapnia on mitochondrial and ER function driving lung injury. Increased CO_2_ levels cause mitochondrial dysfunction, reduce intracellular ATP levels thus altering ER function and promoting misfolding of the Na,K-ATPase protein leading to decreased plasma membrane abundance of the transporter. Hypercapnia-induced dysfunction of the mitochondria and ER may reduce cell reparative potential thus contributing to lung injury. IDH2, isocitrate dehydrogenase 2. Created with BioRender.com.

Apart from ATP, protein folding and formation of disulfide bonds require a specific oxidizing environment of the ER (Araki and Nagata, 2011). The coordinated interaction between glutathione disulfide, hydrogen sulfide, hydrogen peroxide and NO maintains an optimal redox balance in the ER and mediates sulfenylation, sulfhydration and nitrosylation of the folded proteins (Banhegyi et al., 2012; Ellgaard et al., 2018). In addition, oxidative modifications in the ER are reduced by ER-resident oxidoreductases and protein disulfide isomerases, such as ER oxidoreductin 1 (Ero1), protein disulfide-isomerase (PDI), and fumarate reductase 2 (OSM1) (Tu et al., 2000; Araki and Nagata, 2011; Kim et al., 2018). Thus, perturbations of the ER redox balance [by e.g., dithiothreitol (DTT)] cause protein misfolding, activate ER stress and initiate UPR pathways, leading to cellular dysfunction or even cell death (Tatu et al., 1993; Bergmann and Molinari, 2018). Recent evidence suggests that elevated CO_2_ levels alter the oxidizing environment of the ER. Recently, we were able to show that elevated CO_2_ levels induce ER oxidation in hypercapnia-exposed alveolar epithelial cells (Kryvenko et al., 2020). One of the well-characterized types of oxidative protein modification is carbonylation of protein targets. This biochemical reaction is characterized by an irreversible non-enzymatic attachment of carbonyl groups to proteins, which disrupts normal protein folding in the ER by either modifying nascent proteins or by impairing the structure of ER chaperons (England and Cotter, 2004; Dalle-Donne et al., 2006). Interestingly, increased oxidation in the ER leads to ER retention and carbonylation of the Na,K-ATPase β-subunit (Kryvenko et al., 2020, 2021a), a protein that plays a central role in alveolar epithelial junctional function and clearance of alveolar edema, and function of which is impaired in the setting of acute lung injury and hypercapnia (Figure 1; Vadasz et al., 2007, 2008; Kryvenko and Vadasz, 2021). The influence of elevated CO_2_ levels on oxidative processes was also reported in another recent publication in which exposure of human bronchial epithelial cells to hypercapnia led to upregulation of genes involved in cellular responses to oxidative stress pathways (Casalino-Matsuda et al., 2018). Whether hypercapnia affects ER-resident oxidoreductases and protein disulfide isomerases (such as Ero1, PDI, and OSM1) is currently unknown and needs further investigation. Moreover, the ER, redox reactions and iron metabolism are tightly linked together (Banhegyi et al., 2012; Andreini et al., 2018; Hedison and Scrutton, 2019). Therefore, the role of the iron-proteome in CO_2_ sensing and hypercapnia-induced ER oxidation status changes needs further attention.

### Hypercapnia and Endoplasmic Reticulum Calcium Homeostasis

The ER also represents a major calcium storage organelle, which regulates intracellular Ca^2+^ concentrations and oscillations (Krebs et al., 2015). Importantly, Ca^2+^ signaling is centrally involved in several intracellular pathways regulating protein synthesis, cell proliferation, metabolism and apoptosis (Bagur and Hajnoczky, 2017). The protein maturation process in the ER greatly relies on Ca^2+^-dependent chaperons, such as calnexin and calreticulin, two key players in the protein folding cycle (Araki and Nagata, 2011). Physiological Ca^2+^ concentrations are much higher in the ER than in the cytoplasm, which is achieved by sequestering of free Ca^2+^ and the coordinated action of the tissue-specific ATP-dependent Ca^2+^ pumps (SERCA2A and SERCA2B), ER membrane-localized inositol trisphosphate (InsP3R), and ryanodine (RyR) receptors (Schwarz and Blower, 2016; Almanza et al., 2019). Under physiological conditions, a sustained decrease of luminal ER Ca^2+^ levels upon Ca^2+^ release from the ER is prevented by store-operated calcium entry. This process is driven by oligomerization of stromal interaction molecule protein 1 and 2 (STIM1/STIM2) with the plasma-membrane localized calcium release-activated calcium channel protein 1 (ORAI1) and subsequent Ca^2+^ influx into the cell, followed by a SERCA-driven influx into the ER (Schwarz and Blower, 2016; Santulli et al., 2017). Thus, a depletion of the ER Ca^2+^ pool or inactivation of SERCA is associated with ER dysfunction and accumulation of unfolded/misfolded proteins (Sano and Reed, 2013).

**FIGURE 2:**
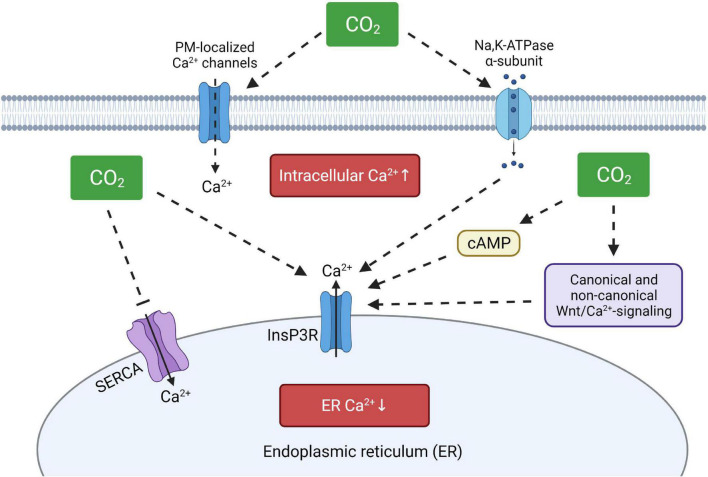
Proposed model of hypercapnia-induced intracellular Ca^2+^disturbances. Elevated CO_2_ levels may increase intracellular calcium concentrations by activation of plasma membrane-localized Ca^2+^ channels, by direct or indirect stimulation of ER-resident InsP3R and by modulating SERCA activity. ER, endoplasmic reticulum; InsP3R, ER membrane-localized inositol trisphosphate receptor; SERCA, sarco/endoplasmic reticulum Ca^2+^-ATPase, cAMP, cyclic adenosine monophosphate. Created with BioRender.com.

Previous studies have reported that hypercapnia promotes elevation of intracellular Ca^2+^ levels in various cell types, thus causing different effects ranging from a decrease of the plasma membrane abundance of specific ion transporters to increased airway contractility (Vadasz et al., 2008; Cook et al., 2012; Turner et al., 2016; Shigemura et al., 2018). Interestingly, both short- and long-term hypercapnia modify intracellular Ca^2+^ levels, suggesting that several sources of the intracellular Ca^2+^ oscillations may exist. Previous reports have shown, that removal of Ca^2+^ from the extracellular medium, treatment with L- and T-type Ca^2+^ channel inhibitors or blocking SERCA activity by thapsigargin are not sufficient to prevent the elevation of intracellular Ca^2+^ concentrations upon hypercapnia, suggesting calcium mobilization from other stores (Nishio et al., 2001; Bouyer et al., 2003). In line with these findings, it was recently shown in alveolar epithelial cells and murine precision cut lung slices that the hypercapnia-induced increase in intracellular Ca^2+^ can be prevented by inhibition of InsP3R, indicating that elevated CO_2_ levels may enhance Ca^2+^ release from the ER (Kryvenko et al., 2021b). These results are also consistent with reports showing that ER-localized InsP3R receptors mediate Ca^2+^ release upon hypercapnia (Cook et al., 2012; Turner et al., 2016). Moreover, increased production of cAMP upon hypercapnia (Lecuona et al., 2013) may additionally stimulate protein kinase A and enhance subsequent release of calcium ions from the ER through InsP3R (Schmidt et al., 2008; Hofer, 2012). In a recent publication, a large-scale transcriptomic analysis of lung, muscle and respiratory cells exposed to hypercapnia revealed upregulation of canonical and non-canonical Wnt signaling pathways, including Fzd9, Wnt7a, Wnt4, and Wnt8b (Shigemura et al., 2019). The non-canonical Wnt/Ca^2+^ signaling cascade is tightly connected to the ER and plays an important role in the regulation of calcium release through InsP3R receptors and is linked to activities of calmodulin kinases activity and protein kinase C, which were previously found to be activated upon hypercapnia (Komiya and Habas, 2008; Vadasz et al., 2008). Interestingly, it has also been found that the Na,K-ATPase, a prominent target of hypercapnia, is involved in Ca^2+^ signaling as well by a direct interaction between the catalytic α-subunit of the Na,K-ATPase and InsP3R, thus modulating Ca^2+^ oscillations (Liu et al., 2008; Aperia et al., 2020). Thus, increasing evidence suggests that the ER is the primary source of increased intracellular Ca^2+^ upon hypercapnia and that enhanced release of Ca^2+^ from the ER may deplete the ER Ca^2+^ stores, which may impair the function of calcium-dependent chaperones, leading to compromised protein folding. These affects might be further aggravated by a marked downregulation of ATP-dependent transporters upon hypercapnia, including SERCA, thus impairing store-operated calcium entry mechanisms (Figure 2).

In addition, previous studies have reported that ER calcium and redox status are interconnected. Activity of RyR and SERCA2b are modified depending on the oxidative status of these molecules (Araki and Nagata, 2011). On the other hand, activation of InsP3R receptors and subsequent release of Ca^2+^ leads to a hyperoxidizing ER environment and apoptosis *via* CCAAT/enhancer-binding protein-homologous protein (CHOP) (Li et al., 2009). Thus, it may well be that a decrease in ATP production and increased ER protein oxidation upon elevated CO_2_ levels also contribute to alterations in ER Ca^2+^ homeostasis upon hypercapnia.

## Hypercapnia, Endoplasmic Reticulum Stress and Adaptive vs. Maladaptive Unfolded Protein Response

A consequence of protein misfolding/unfolding in the ER, is ER stress and subsequent activation of IRE1α-, PERK-, and ATF6-mediated UPR pathways. The UPR response may be adaptive or maladaptive, depending on the markedness and duration of the stimulus (Wang and Kaufman, 2016). The adaptive mechanisms “aim” to restore the protein folding homeostasis in the ER by downregulating protein synthesis, activating ERAD and modulating function of specific ER chaperones. If the initial UPR response does not allow coping with ER stress, the maladaptive arm of UPR will be activated that may lead to cellular death, mostly *via* apoptosis (Wang and Kaufman, 2016).

A numbers of studies have shown that physiological ER stressors selectively activate UPR branches, thereby triggering non-classical stress responses within the ER, which do not lead to cellular death and have rather adaptive character (Raina et al., 2014; Bergmann et al., 2018). In line with this notion, we now know that exposure of alveolar epithelial cells to hypercapnia transiently activates IRE1α and induces ERAD of the ER-resident β-subunit of the Na,K-ATPase, thereby decreasing plasma membrane abundance of the transporter (Kryvenko et al., 2021b). Furthermore, enhanced protein degradation in the ER by ERAD is associated with increased ubiquitination of the target protein, which has been shown to occur upon hypercapnia (Gwozdzinska et al., 2017). Of note, a recent study identified the IRE1α interacting partner, TNF receptor-associated factor 2 (TRAF2), as a novel E3-ligase involved in the polyubiquitination of the Na,K-ATPase β-subunit (Gabrielli et al., 2021). However, whether TRAF2 is additionally required for ERAD of the Na,K-ATPase will need to be addressed in future studies. Interestingly, treatment of cells with CO_2_ levels of up to 120 mmHg for a duration of 5 days is not associated with increased apoptosis or cellular death in alveolar epithelial or mesenchymal cells (Vohwinkel et al., 2011), suggesting that at least in these settings of hypercapnia a rather adaptive type of UPR is activated.

It is well documented that elevated CO_2_ levels initiate specific signaling cascades in cells, including activation of ERK1/2, JNK, and AMPK-α_1_ that drive retrieval of the Na,K-ATPase and epithelial sodium channel (ENaC) from the plasma membrane, thereby causing alveolar epithelial barrier dysfunction and altering alveolar fluid balance (Vadasz et al., 2008; Welch et al., 2010; Gwozdzinska et al., 2017). Moreover, exposure of skeletal muscles to increased CO_2_ concentrations leads to stimulation of AMPK-α_2_ and is associated with a decrease in protein synthesis and increased muscles catabolism (Jaitovich et al., 2015; Ceco et al., 2017; Korponay et al., 2019). In general, AMPK activation is a response to metabolic stress by sensing AMP:ATP and ADP:ATP ratios, aiming to reestablish energy balance by reducing anabolic processes that require ATP and by promoting catabolic mechanisms that generate ATP (Garcia and Shaw, 2017). In contrast, in the setting of short-term hypercapnia, AMPK activation is independent of the metabolic status of the cell and is rather secondary to intracellular Ca^2+^ signaling (Vadasz et al., 2008). Notably, knockdown of AMPK in bronchial epithelial cells leads to a significant increase in CHOP levels resulting in ER stress and apoptosis (Liu et al., 2018). Moreover, AMPK activation downregulates BiP levels induced by tunicamycin or thapsigargin and has been found to regulate ER and mitochondrial morphology upon stress conditions, thus preventing mitochondrial fragmentation and apoptosis (Wikstrom et al., 2013; Kim et al., 2015).

Extracellular signal-regulated kinase, a member of the mitogen-activated protein kinase (MAPK) family, has been shown to play an essential role in UPR by interacting with IRE1α and by promoting transcription of pro-survival anti-apoptotic proteins, such as myeloid leukemia cell differentiation protein-1 (Mcl-1), Bcl-2 and B-cell lymphoma-extra large protein (Bcl-xL) (Darling and Cook, 2014). Furthermore, activation of ERK1/2 has been shown to be cytoprotective upon ER stress, by downregulating cellular apoptosis upon thapsigargin- and tunicamycin-induced UPR (Arai et al., 2004; Hu et al., 2004).

In addition, several other mechanisms may contribute to the adaptive or maladaptive signals upon hypercapnia. For example, hypercapnia has been found to inhibit autophagy in human macrophages by increasing expression of Bcl-2 and Bcl-xL, thus blocking Beclin-1 apoptotic complex formation (Casalino-Matsuda et al., 2015). Of note, Bcl-2 is involved in the regulation of ER calcium homeostasis und upregulation of the molecule may play a protective role upon ER stress by lowering steady-state levels of ER Ca^2+^ *via* InsP3R activation (Sano and Reed, 2013). However, the anti-apoptotic effects of Bcl-2 are inhibited by JNK (Sano and Reed, 2013) that is markedly upregulated in the setting of acute and chronic hypercapnia (Vadasz et al., 2012a; Dada et al., 2015; Gwozdzinska et al., 2017). In fact, the role of activated JNK, in contrast to AMPK and ERK1/2, is usually associated with an enhanced pro-apoptotic ER stress response. On the other hand, JNK is involved in the downstream cascade of IRE1α activation, the UPR branch responsible for preventing ER overload by ERAD (Maurel et al., 2014; Prischi et al., 2014; Almanza et al., 2019). Recently, hypercapnia has been associated with increased airway smooth muscle contractility in the setting of asthma, which is mediated by activation of caspase-7, an apoptosis-related cysteine peptidase (Shigemura et al., 2018). Interestingly, caspase-7 has also been found to be involved in the ER-stress mediated cell death upon thapsigargin treatment and caspase-7 ablation was able to reprogram the UPR and reduced JNK-induced apoptosis (Dahmer, 2005; Choudhury et al., 2013).

Thus, while activation of AMPK, ERK1/2, JNK, and caspase-7 drive clearly deleterious (maladaptive) signals leading to cellular dysfunction upon hypercapnia, activation of these signaling molecules may, at least in part, limit further injury by reducing the elevated CO_2_-induced ER stress, as part of an adaptive mechanism.

## Conclusion

**FIGURE 3:**
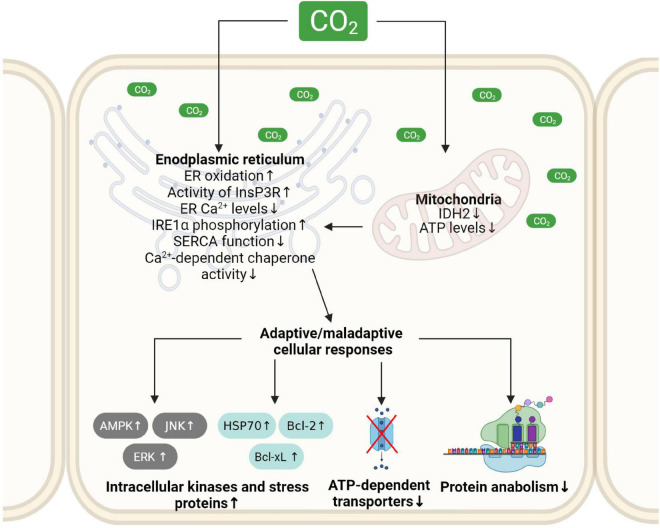
Schematic depiction of molecular mechanisms driving hypercapnia-induced ER dysfunction and subsequent adaptive and maladaptive cellular responses. Elevated CO_2_ levels reduce cellular ATP production, impair the oxidizing environment and alter calcium levels in the ER. These alterations in ER homeostasis cause ER stress and initiate adaptive and maladaptive cellular responses. ER, endoplasmic reticulum; IDH2, isocitrate dehydrogenase 2; InsP3R, ER membrane-localized inositol trisphosphate receptor; JNK, c-Jun N-terminal kinase; ERK1, extracellular signal-regulated kinase; AMPK, AMP-activated protein kinase; Bcl2, B-cell lymphoma 2 protein; Bcl-xL, B-cell lymphoma-extra large protein; HSP70, heat shock protein 70. Created with BioRender.com.

Protein maturation and folding in the ER require a specific milieu, which depends on Ca^2+^, ATP and an oxidative environment. Recent studies focusing on the pathophysiological effects of hypercapnia established that elevated CO_2_ levels alter the ER folding machinery. The molecular mechanisms driving ER dysfunction upon high CO_2_ concentrations include reduced cellular ATP levels, a Ca^2+^ disbalance in the ER, as well as altered redox homeostasis of the organelle (Figure 3). These events lead to ER stress, UPR, and ERAD of target proteins, potentially resulting in tissue and organ malfunction. To what extent these signals are adaptive or maladaptive depend on the extent and duration of hypercapnia and require further experimental assessment.

## Author Contributions

VK and IV drafted, edited, and approved final version of the manuscript.

## Conflict of Interest

The authors declare that the research was conducted in the absence of any commercial or financial relationships that could be construed as a potential conflict of interest.

## Publisher’s Note

All claims expressed in this article are solely those of the authors and do not necessarily represent those of their affiliated organizations, or those of the publisher, the editors and the reviewers. Any product that may be evaluated in this article, or claim that may be made by its manufacturer, is not guaranteed or endorsed by the publisher.
